# Of men in mice: the development and application of a humanized gnotobiotic mouse model for microbiome therapeutics

**DOI:** 10.1038/s12276-020-0473-2

**Published:** 2020-09-10

**Authors:** John Chulhoon Park, Sin-Hyeog Im

**Affiliations:** 1 Department of Life Sciences, Division of Integrative Biosciences and Biotechnology, Pohang University of Science and Technology, Pohang, Gyeongbuk 37673 Republic of Korea; 2 ImmunoBiome Inc. POSTECH Biotech Center, Pohang, 37673 Republic of Korea

**Keywords:** Experimental models of disease, Translational research

## Abstract

Considerable evidence points to the critical role of the gut microbiota in physiology and disease. The administration of live microbes as a therapeutic modality is increasingly being considered. However, key questions such as how to identify candidate microorganisms and which preclinical models are relevant to recapitulate human microbiota remain largely unanswered. The establishment of a humanized gnotobiotic mouse model through the fecal microbiota transplantation of human feces into germ-free mice provides an innovative and powerful tool to mimic the human microbial system. However, numerous considerations are required in designing such a model, as various elements, ranging from the factors pertaining to human donors to the mouse genetic background, affect how microbes colonize the gut. Thus, it is critical to match the murine context to that of human donors to provide a continuous and faithful progression of human flora in mice. This is of even greater importance when the need for accuracy and reproducibility across global research groups are taken into account. Here, we review the key factors that affect the formulation of a humanized mouse model representative of the human gut flora and propose several approaches as to how researchers can effectively design such models for clinical relevance.

## Gut microbiome: making a human model in mice

Gut microbiota play important roles in health and disease, and now the effects of specific microbes sourced from humans can be tested in germ-free mice, which have no microbiome of their own. Many factors can affect how well the transplanted microbiome will reflect the human microbiome it is meant to mimic. Sin-Hyeog Im and John Chulhoon Park of POSTECH in South Korea have reviewed key factors that affect donor microbiomes and how well they transplant to mouse models. Diet, socioeconomic background, ethnicity, and exercise regime affect the human source microbiome. Differences in mouse and human anatomies, murine genetic and immunological backgrounds, and the conditions under which the mice are reared can affect how faithfully the transplanted microbiome reflects its human source. This review will help in designing more translatable animal models to test microbial-based therapies.

## Introduction

From the discovery of bacteria in the late 17th century to the modern understanding of their intricate roles in human physiology, the study of microbes in the human context has progressed considerably. A significant source of research advancement has come through the development of germ-free (GF) mice, which lack all microorganisms, effectively allowing the transfer of selective bacterial species or whole fecal microbiota^1,2^. Although antibiotic-treated mice can attempt to mimic GF conditions, the elimination of microbes is neither complete nor free of off-target effects^3^. This was specifically illustrated in two publications by Yongwon Choi’s group, in which enteritis was observed in a gut microbiota-dependent manner in one strain of antibiotic-treated mice^4^. However, when the model was later placed under GF conditions, opposing results were observed, indicating that the previous findings were the outcome of off-target effects of the antibiotics rather than those directly of the gut microbiota^5^. GF models, on the other hand, serve as a completely blank microbial background to offer an invaluable opportunity to see not only correlative relationships but also causal effects through the association of gut microbes with the host^3^. This strategy has paved a new path for pinpoint analysis of the taxa-specific capacity of microbes to regulate host functions ranging from immune tolerance and metabolism to neurological and endocrine modulation^6–10^.

The capabilities of GF mice have further been demonstrated through fecal microbiota transplantation (FMT), in which fecal samples of donors are transplanted into the guts of recipients. FMT was first proposed in 1958, and the concept was strengthened in 1983 with the treatment of *Clostridioides difficile*-induced pseudomembranous enterocolitis using the transfer of fecal enema, thus highlighting the ability of the transplanted microbiota to modulate host physiology and even alleviate disease^11,12^. Although gnotobiosis has been utilized in mice since 1959, it was not until the 1980s when FMT of human feces was applied to create a humanized gnotobiotic mouse model, which has since been a revolutionary strategy to formulate in vivo systems of the human flora^13–17^. The colonization of human gut microbes into GF murine disease models has recently been used to elucidate the influence of human gut microbes on cancer immunity, autoimmunity, and even malnutrition among children^6,7,18–20^. These studies clearly demonstrate that humanized gnotobiotic mouse models have contributed to the advancements in biomedicine, bridging the gap between human and animal gut physiology and pathology. However, inadequate understanding on the full intricacies of the gut microbiota and lack of standardized protocols in creating humanized gnotobiotic mouse models place major constraints on the effective translatability of the system. In this review, we discuss the core foundations of the humanized gnotobiotic mouse model, taking into consideration the selection criteria for human donors, the translatability of the human gut microbiota into murine settings, and the necessary points of optimization for relevant applications.

## Where are the fecal samples from?

Humanized gnotobiotic mouse models are innovative in their ability to integrate the human microbiota into the murine system through FMT. However, a major caveat in design comes from the variances in the input microbiota from human donor feces, depending on from whom the sample is derived, which will inevitably result in different outcomes in the mouse model. Such variations can be an issue for basic science that aims to produce ubiquitous findings, making the standardization of donor feces critical. Hence, it is necessary to identify factors that affect the human gut flora.

Jeffrey I. Gordon’s group has extensively reported cross-site and regional case studies in the Amazonas of Venezuela, rural Malawi, Bangladesh, and the United States to identify the significant variations in the human microbiota composition in both children and adults, characterized by distinct clustering of taxa, depending on the region (Table 1)^7,20–25^. Particularly, non-western children have higher flora diversity and Bacteroidetes levels with lower Firmicutes levels than western children, and *Prevotella* may be a specific discriminatory taxa between these regions^21,26,27^. Likewise, in comparing the gut microbiomes of East Asian countries (China, Japan, and Korea) to those of the United States, Nam et al.^28^ revealed that the levels of Firmicutes were higher among Americans. Yet interestingly, variations among the Asian groups were also found, as the Japanese group had a higher abundance of Actinobacteria, *Bifidobacterium*, and *Clostridium*, whereas the Koreans and Chinese groups had a higher abundance of Bacteroidetes but different levels of *Bacteroides*, *Clostridium, Prevotella*, and *Faecalibacterium*^28^. These differences in bacterial diversity and abundance across regions highlight the influence of geographical factors on the gut microbiota; however, a closer examination brings to light further specific variables into the equation.

**Table 1:** Factors affecting the human gut microbiota.

Factor	Impact on the gut microbiota	Ref.
*Donor geography*		
USA, Malawi, Amazonas of Venezuela	Large variation in composition between western and non-western populations Malawian and Venezuelan children have higher flora diversity than American children Distinct flora characteristics between Malawian and Venezuelan children	^21^
Italy, Burkina Faso	*Prevotella* and *Xylanibacter* as discriminatory taxa for western vs non-western children Bacteroidetes were enriched and Firmicutes were depleted in non-western children	^26^
Italy, Tanzania	Higher flora diversity and richness among Tanzanians, with enriched *Bifidobacterium*, *Prevotella, Treponema*, and unique clustering of *Clostridiales* members	^30^
Peru, USA	Higher flora diversity among Peruvians Proteobacteria, Spirochetes, and *Prevotella* were enriched in Peruvians Actinobacteria, *Bacteroides, Blautia*, and *Dorea* were enriched in Americans	^31^
Bangladesh, USA	Bangladeshi children have higher flora diversity; *Prevotella, Butyrivibrio*, and *Oscillospira* were enriched, but *Bacteroides* were depleted	^27^
Korea, Japan, China, USA	USA: highest Firmicutes Japan: highest Actinobacteria*, Bifidobacterium*, and *Clostridium* China: highest Bacteroidetes, *Bacteroides*, lowest *Clostridium* Korea: highest *Prevotella* and *Faecalibacterium*	^28^
*Donor ethnicity*		
America (African American, Asian and Pacific Islander, Caucasian, Hispanic)	12 microbial taxa differ in abundance: *Peptococcaceae, Dehalobacteriaceae, Christensenellaceae*, Clostridiales*, Veillonella*, RF39, *Verrucomicrobiaceae, Victivallaceae, Odoribacteriaceae, Odoribacter, Rikenellaceae, Coriobacteriaceae*	^32^
Netherlands (Moroccan, Turk, Ghanaian, Dutch)	*Prevotella* were enriched in Moroccans, Turks, and Ghanaians *Bacteroides* were enriched in African and Asian Surinamese populations; least microbial diversity *Clostridiales* were enriched in the Dutch population; greatest microbial diversity	^35^
*Donor age*	Microbiota composition “matures” as children develop, normally after 3 years of age Bacterial diversity increases with age during childhood Undernourished children have “immature” gut flora compared with the progression of composition evolution of healthy children	^7^^,^^20^^,^^21^^,^^22^^,^^23^
*Diet*		
Undernutrition	“Immature” microbial floras in undernourished children Altered metabolic pathways Up to 220 taxa differ in abundance *Ruminococcus gnavus* and *Clostridium symbiosum* prevent growth impairments	^7^^,^^20^^,^^22^
Subsistence mode	The presence of the parasite *Entamoeba* leads to higher flora diversity Hunter-gatherers have higher Proteobacteria and *Succinivibrionaceae*, but lower Firmicutes and *Ruminococcus* Fishing populations have higher *Bifidobacterium* but lower *Bacteroidales*	^29^^,^^30^^,^^31^
High-fat	Increase in F/B ratio, *Alistipes, Bilophila, Bacteroides, Lactococcus lactis, Pediococcus acidilactici, Streptococcus thermophilus,* and *Staphylococcus* and a decrease in Firmicutes such as *Roseburia, Eubacterium rectale, Ruminococcus bromii*	^36^^,^^37^^,^^42^
High-fiber	Decrease in F/B ratio Enrichment in *Bifidobacterium* and *Lactobacillus* Decrease in *Prevotella*	^44^^,^^48^^,^^49^
*Exercise*		
	Enlargement of cecum, increase in n-butylate concentration, decrease in intestinal inflammation	^53^^,^^54^
	Increase in Bacteroidetes (*S24-7*) and decrease in Firmicutes and Actinobacteria	^55^^,^^56^
	Enrichment in Firmicutes (*Allobacterium* and *Clostridiales*), presence of *Faecalibacterium prausnitzii*	^54^^,^^57^

The diversity of gut microbiota compositions among children of varying socioeconomic backgrounds within the same geographical contexts indicates another major source of impact on the gut flora—that of cultural, economic, and dietary practices (Table 1)^20–24^. A study comparing subsistence mode-distinct populations in southwestern Cameroon more closely illustrates this rationale by showing that even within a small geographic range, populations harbor distinct gut floras depending on the presence of *Entamoeba* parasites and whether the subsistence mode is hunter-gatherers or farmers^29^. The finding that the presence of *Entamoeba* correlates with higher alpha diversity and that hunter-gatherers harbor a unique microbial signature parallels similar studies conducted among the Hadza hunter-gatherers of Tanzania and the Matses of Peru^29–31^. In these groups, Proteobacteria and *Lachnospiraceae uncl* as well as *Succinivibrio* and *Ruminobacter* species were all enriched among hunter-gatherers, who also had higher microbial richness and biodiversity compared with their urban counterparts in the United States and Italy^29–31^. This may be of particular interest for groups identifying singular microbes for therapeutic applications, as studying the gut floras of populations with more-diverse microbial compositions may lead to more representative candidate selection. In addition, the socioeconomic consequences on health, markedly undernutrition among children, result in the underdevelopment of the gut flora compared with those of their healthy regional counterparts, leading to both a less diverse and “immature” flora, as defined by age-associated compositions^20,22–24^. The fact that dietary treatments for undernutrition cannot fully normalize the gut flora highlights the complex and long-lasting consequences of the environmental and socioeconomic impacts on the gut flora^22,23^.

The ethnicity of people within the same population contributes to further variations in the microbiome. Among humans, the idea of a “core” microbiome does exist, as studies have indicated anywhere between 12 and 25 shared taxa, which vary in abundance, that can be heritable across generations and correlate with disease probabilities for specific ethnic groups^32–35^. However, there still lie clear distinctions in the specificity of the makeup of the gut flora across populations (Table 1). A recent study conducted in the urban Netherlands revealed that even within the “core” microbiome of the population, ethnic-specific profiles exist^35^. Another study with 1673 individuals of varying ethnicities across the US further found ethnicity to be a more representative factor in distinguishing microbiota compositions than BMI, age, and sex—and enough so that the compositional differences could even partly predict corresponding ethnic groups^32^. This suggests that not all humanized murine microbiota models may accurately portray different populations, emphasizing the need to consider from whom fecal samples are collected for the representative studies.

### Influence of the human donor’s diet and exercise on the gut microbiota

The significant impact of dietary factors on the gut flora has been well regarded and must be further examined to select the appropriate human fecal donors. The effects of a high-fat diet on the gut flora are among the most extensively published in the field, showing drastic shifts in microbial signatures that are most identifiable by the sharp increase in the Firmicutes to Bacteroidetes (F/B) ratio (Table 1)^36,37^. The F/B ratio is significant owing to its correlations with conditions such as obesity, microbiota maturation, dysbiosis, and systemic inflammation^38–40^. Many physiological disorders are associated with high-fat feeding, but rather than a direct manifestation of the diet itself, evidence point to the microbiota as a key determinant, as bacteria such as *Akkermansia muciniphila*, a major mucus layer-residing bacterium in healthy humans, can reverse diet-induced obesity and diabetes^41^. In human studies, short-term diets composed of purely animal products have led to an increase in bile-tolerant microbes, such as *Alistipes, Bilophila*, and *Bacteroides*, with decreases in *Roseburia, Eubacterium rectale, Ruminococcus bromii*, and other Firmicutes that utilize dietary plant polysaccharides^42^. Animal product-based diets also induced higher colonization of lactic acid bacteria such as *Lactococcus lactis, Pediococcus acidilactici*, and *Streptococcus thermophilus*, as well as several *Staphylococcus* species and fungi^42^. The dysbiosis brought upon by animal-based diets may correlate with higher incidences of inflammatory bowel disease, often marked by an increase in *Bilophila wadsworthia* levels in mice kept on a high-fat diet^42,43^. On the other hand, short-term high-fiber and plant-based diets induce *Clostridium*, *Methanobrevibacter, Bifidobacterium*, and *Lachnospiraceae* and decrease *Prevotella*^44^. Interestingly, with long-term vegan or vegetarian-based diets, lower levels of *Prevotella* are maintained, and the levels of *Clostridium* cluster XIV, *Bifidobacterium, Bacteroides sp*., and *Enterobacteriaceae* are decreased compared with those in omnivores^45–47^. These contrasting observations may highlight the varying impact of the duration of dietary patterns on reshaping the gut flora, but also reflect the regional and ethnic influences across studies. Fiber particularly influences the gut microbiota by acting as a microbiota-accessible carbohydrate, with high dietary fiber increasing *Bifidobacterium* and *Lactobacillus* levels and lowering the F/B ratio, whereas low-fiber consumption leads to depletion of microbial diversity and higher *Bacteroides*^26,48–50^. The importance of the type of dietary fiber was recently demonstrated by Deehan et al.^51^; small discrete alterations in the chemical structure of fibers caused distinct enrichments of certain taxa, leading to altered metabolite output. More specifically, soluble fibers increase the alpha diversity and relative abundance of Proteobacteria and Actinobacteria, whereas insoluble fibers increase Bacteroidetes and Euryarchaeota, as well as IgA and tumor necrosis factor-alpha levels^52^. Thus, both human donor and murine recipient diets must be carefully assessed for desirable FMT outcomes.

Exercise patterns must also be evaluated as another indicator of health status. Increasing evidence suggests that exercise frequency leads to alterations in the gut flora (Table 1). Exercise in murine models has been shown to cause shifts in the gut physiology and microbiota composition, leading to enlargements of the cecum, modulation of intestinal villi, increased n-butyrate concentrations, and reduced intestinal inflammation^53–55^. However, the exact impact of exercise on the gut flora is contested, as demonstrated by two studies utilizing high-fat diet-induced obese mice with or without exercise^54,55^. Evans et al.^55^ reported that exercise increases Bacteroidetes, particularly the *S24-7* family, and decreases Firmicutes and Actinobacteria, although the class Clostridia observed elevated levels for *Clostridiaceae, Lachnospiraceae*, and *Ruminococcaceae* in the exercise groups. This increase in Bacteroidetes following exercise was further supported by Carbajo-Pescador and colleagues^56^. However, the data by Campbell et al. showed that Firmicutes, principally *Allobaculum* and *Clostridiales*, are enriched, with *Faecalibacterium prausnitzii* being unique to the exercise groups^54^. This was bolstered by Lambert and colleagues, who also showed increased levels of Firmicutes and *Bifidobacterium*, whereas the levels of *Bacteroides/Prevotella* were lowered following exercise^57^. In humans, Allen et al.^58^ demonstrated minor, but statistically significant, increases in several Firmicutes genera and decreases in two Bacteroidetes genera, including *Bacteroides.* Yet, a similar study conducted by Kern et al.^59^ did not observe significant differences. It is suspected that experimental designs caused significant variations in the outcomes of these studies, which warrants more in-depth inquiries into the effects of exercise patterns prior to donor selection for FMT utilization.

With so many factors acting upon the composition of the human gut microbiome, research groups must assess how their experimental systems will be designed. As described above, the diverse sources attributed to unique microbial signatures can lead to well-designed correlative studies that compare the gut floras and resulting host phenotypes of different populations. However, research that aims to uncover the mechanistic relationships of microbes that apply to the general physiology and pathology of humans needs to look toward an unbiased source for the gut microbiota. Although it is difficult to define a singular “healthy” or “normal” gut microbiota composition, researchers could take these factors into consideration as they determine which source is most appropriate in representing the underlying human biology of microbe–host interactions. These efforts will be of further importance in generating results that are reproducible and relevant across different study sites and investigators.

### Human and murine anatomy

Much of modern-day gut microbiota research is moving toward the use of GF mice for its translatability. However, the use of GF mice must be scrutinized in light of the biological differences between mice and humans. Although the general anatomy of mice and humans is similar, distinctions remain in regard to the structural design of the gastrointestinal tract. The proportionally larger colon and cecum surface area and taller intestinal villi in mice may function to increase nutrient uptake and essential element production and subsequently harbor a larger microbial flora^60–62^. In addition, variations in the microbiota composition may arise owing to the appendix in humans, a vestigial organ acting as a microbial reservoir that mice lack^63^. Indeed, although mice are known to have “cecal lymphoid patches” that may be synonymous with the human appendix, the flora of these two compartments differ, as the human appendix is dominated by Firmicutes, Proteobacteria, Bacteroidetes, and Fusobacteria, respective to abundance, whereas murine cecal lymphoid patches consist of Bacteroidetes, Firmicutes, Actinobacteria, and Proteobacteria, respectively^64,65^. These distinctions denote a larger divergence in the gut flora characteristic of each model, prompting the need to ask how much of the human microbiome can mice hold.

### The gut microbiota compositions of humans and mice

Human and murine gut floras share 90% and 89% similarities in phyla and genera, respectively^66^. Although these numbers may seem indicative of high gut microbiota resemblance, a closer look reveals key discrepancies in the microbial signatures, particularly in regard to the makeup and abundance of microbes. The most discernible difference is the ratio of the two major phyla, with humans having a greater F/B ratio, whereas the inverse is true for mice^64–68^. In mice, the phylum Bacteroidetes is mainly composed of the *S24-7* family, and Firmicutes consists of *Clostridiales*^67^. On the other hand, in humans, Bacteroidetes is mainly composed of *Bacteroidaceae*, *Prevotellaceae,* and Firmicutes of the *Ruminococcaceae* family^67^. In total, the top 15 genera found in mice and humans vary by the presence or absence of five genera, and the relative abundance of the top genera deviates drastically; mice have 44.7% *S24-7*, 25.3% *Clostridiales*, and 5.0% *Oscillospira*, whereas humans have 27.5% *Bacteroides*, 10.2% *Ruminococcaceae*, and 9.7% *Clostridiales*^67^. In addition, humans and mice each carry exclusive genera, with humans harboring *Faecalibacterium, Mitsuokellla, Megasphera, Dialister, Asteroleplasma, Succinivibrio, Sutterella, Paraprevotella*, and *Phascolarctobacterium*, whereas *Mucispirillum* is found primarily in rodents^66^.

The presence of unique microbes between humans and mice may pose a limitation on the generation of humanized gnotobiotic mouse models, particularly if these bacteria have host-specific physiological influences; for example, murine-segmented filamentous bacteria (SFB). Chung et al.^69^ revealed that immune maturation after cross-species FMT is host dependent, as only mouse microbiota transplants, but neither those of human nor rat, could induce immune cell expansions in GF mice. Only after colonization with SFB could a partial expansion in CD4^+^ T cells occur in humanized GF mice, highlighting a host-specific requirement following interspecific FMT^69^. This is in line with previous reports on the role of SFBs in innate immunity induction in murine contexts and may be indicative of diverging mechanisms of immune maturation among animal species^6,70^. Humans and mice have a distinct composition of peripheral immune systems, with human blood being composed of more neutrophils than lymphocytes (50–62% neutrophils to 30–50% lymphocytes), whereas murine peripheral leukocytes are composed mostly of lymphocytes (75–90% lymphocytes to 10–25% neutrophils)^71–73^. It is thereby unclear whether the differences in human and mouse gut microbiotas result in the peripheral immune makeup or whether the immune system itself is a key host-specific factor regulating the colonization of transplanted microbiota. Humanized gnotobiotic immunocompromised mouse models can serve as a blank slate for both microbial and immunological transplants, hence presenting a novel opportunity to shed more light on this relationship.

### Microbial metabolite production in humans and mice

A key role of gut bacteria is to aid in the digestion of food and the generation of micronutrients that our bodies cannot synthesize alone. In mice, digestion is a process that takes between 6 and 8 hours—almost tenfold shorter when compared with humans^74,75^. This parallels the much quicker basal metabolic rate and energy expenditure in mice^75,76^. The higher turnover of digested foods may result in a divergence in gut microbe behavior between mice and humans. The concept of the “mucus-trap” in mice, in which furrows in the proximal colon recycle bacteria within the mucus back into the cecum, and coprophagy are mechanisms utilized to maximize nutrient uptake and microbial efficiency^77,78^. Consequently, measures of fecal lactate and short-chain fatty acids (SCFAs) indicate that the gut microbiota of mice generates higher concentrations of lactate than that of humans, although humans still produce higher levels of some SCFAs, such as acetate and propionate^68^. This is consistent with previous findings correlating higher F/B ratios with elevated acetate levels in both humans and mice, particularly during obesity, as well as those demonstrating that Firmicutes are acetate producers^38,68,79–82^. Furthermore, the elevated levels of lactate in mice may be partly owing to the greater presence of lactate-producing bacteria, such as *Lactobacillus*, but it may also be further indicative of differences in flora characteristics between the species. Lactate produced in the intestines is often converted into SCFAs, such as acetate and propionate, by lactate-fermenting bacteria and is generally only detected in human feces during gastrointestinal diseases^83–86^. SCFAs have recently gained much attention owing to their ability to regulate host immune responses, notably with butyrate promoting regulatory T-cell (Treg) differentiation to enhance immune regulation^87–89^. Furthermore, acetate has been found to protect mice from DSS-induced colitis by elevating ROS levels in neutrophils to cause apoptosis^90^.

### Murine genetic and immunological backgrounds

Beyond interspecies distinctions between mice and humans, microbial phylogenetic differences exist across murine genetic backgrounds. Numerous reports have indicated a general trend in which BALB/c mice have greater microbial diversity than mice of other strains, including C57BL/6 mice (Table 2)^66,91–99^. These differences were particularly evident when comparing F/B ratios, which were higher in BALB/c mice, as well as in the compositional variations at the genus and family levels^66,91,94,96,97^. The phyla of modified strains, particularly immunocompromised mice such as nonobese diabetic (NOD) or severe combined immunodeficient mice (SCID), can even vary up to 10% with BALB/c mice, and while cohousing and FMT procedures can compensate for the compositional differences, they produce neither complete nor lasting effects^66,95,96,98,99^. Interestingly, mating BALB/c and C57BL/6 mice results in pups that harbor distinct microbial signatures from those of the parents but are similar among one another, indicating a significant role of genetic background on the gut flora that can be generationally conferred^99^.

**Table 2:** Factors affecting microbiota colonization in humanized murine gut flora models.

Factors	Impact on the gut microbiota	Ref.
*Genetic background*		
C57BL/6	Higher Bacteroidetes, *Lactobacillus*, and *S24-7* More susceptible to microbiota disruptions	^91^^,^^95^^,^^107^
BALB/c	Higher diversity, higher F/B ratio, higher IgA levels	^91^^,^^93^^,^^96^
Immunodeficient	Lower diversity, higher SCFA production, better FMT colonization	^98^^,^^100^^,^^101^^,^^106^
*Microbiota succession*		
	83% and 73% of taxa at the class and genus levels are transmitted to FMT recipient offspring	^36^
	Gut microbiota resemblance between parents and offspring can last up to 21 weeks	^106^
	Mouse genetic background can alter microbiota transmission	^106^^,^^107^
*Sample preparation*		
Fresh	Fresh sample usage may result in a flora composition most representative of the donor	^109^
	Storage at 37 °C for >24 h leads to alterations in the composition of the sample	^109^
Frozen	In mice, frozen sample transplants initially lead to low diversity after colonization but eventually stabilize after 7 days	^36^
	Cryopreservation may significantly lower the amount of viable bacteria	^109^
	Freezing may alter the F/B ratio	^113^
	If freezing is necessary, it should be done at −80 °C with maltodextrin-trehalose solution and thawed quickly at 37 °C	^109^
*Delivery route*		
	Rectal administration correlates with better transplant outcomes in clinical settings	^123^^,^^124^
	Rectal delivery can reduce sepsis occurrences Oral gavage may be easier and more convenient	^125^

One explanation for the mechanism is related to the differences in immunological characteristics induced by the genetic background. Fransen et al.^96^ demonstrated that BALB/c mice have naturally more abundant and diverse immunoglobulin A (IgA) than C57BL/6 mice, and this essential bacteria-associating antibody is responsible for the variance in the gut flora composition. The differences between BALB/c and SCID mice further highlight the role of innate immunity in regulating the composition of the gut microbiota. It is well established that gut microbes direct immunological development, which in turn functions to maintain gut flora and mucosal homeostasis^100^. It is interesting to note that immunodeficient mice have higher levels of *Lactobacillus*, which often induces innate immunity^98,100,101^. Correspondingly, the gut flora of immunodeficient mice also produce greater levels of SCFAs, which have antiinflammatory, epithelial, and mucus lining-regulating, and microbiota composition-modulating functions^101–104^. These characteristics of immunodeficient strains may indicate possible mechanisms of compensating for the compromised immune system. Genetic and immunological settings in murine systems can thus direct the compositional makeup of the gut flora. In designing biomedical studies using humanized gnotobiotic mouse models, careful attention will be required in selecting the appropriate mouse strains to reflect both the aims of the study and ability of the microbes to colonize the gut.

### Vertical transmission of microbiota in GF mice

The concept of microbiota transference during vaginal birth has been a long-established observation, defining the modern understanding of vertical microbiota succession^105^. Although intergenerational flora movement has been well recorded within the same species, it is yet unclear whether the transplanted human microbiota can be passed down to subsequent generations in the murine context. However, emerging evidence indicates that transplanted flora can transfer onto murine offspring without significant loss of diversity and that even up to 83% and 73% of taxa at the class and genus levels, respectively, are shared between the first- and second-generation offspring (Table 2)^36^. A similar finding showed that GF pregnant mice inoculated with the microbiota from control or antibiotic-treated mice have a high microbiota resemblance in the offspring that lasts up to 21 weeks after birth^106^. However, from these studies, a few points of consideration are also apparent, namely, that *IL-10*^*−/−*^ immunodeficient mice are more permissive to microbiota succession, antibiotic-treated donor microbiota lead to taxa loss and instability in recipient offspring, and the effect of the recipient offspring diet eventually overcomes the passed down microbiota^36,106^. Buhnik-Rosenblau et al. investigated the differing levels of *Lactobacillus johnsonii* between C57BL/6 and BALB/c mice^107^. The abundance of this microbe was high in both the parents and offspring of C57BL/6, whereas it was significantly lower in BALB/c strains and unable to be colonized after FMT^107^. However, upon crossing, the C57BL/6 ✕ BALB/c offspring contained intermediate levels of *L. johnsonii*, indicating the role of genetic background in dictating how and which gut microbes are colonized^107^. Further demonstration of vertical transmission in mice has been shown in an immunodeficient murine colitis model, where the protective effect of antibiotics could be passed down to the immunodeficient offspring, and conversely, pups genetically resistant to the model could acquire colitis upon cross-fostering with immunodeficient mothers^108^. Hence, although vertical transmission of microbiota in the murine context occurs, there are several factors that must be taken into consideration, and for multigenerational studies, researchers will have to determine whether fresh transplants will be required for every generation.

### Fecal sample preparation

Methods for stool sample preparation further complicate how transferred gut microbiota develop within humanized gnotobiotic mouse models. Although the utilization of FMT for clinical and research purposes is becoming increasingly popular, there is yet to be a standardized protocol for preparing and handling fecal samples. With sparse longitudinal research on the effects of sample handling on interspecific FMT, it is currently difficult to fathom the extent of variation between institutions and their procedures, leading to doubt on how congruent FMT-utilizing studies actually are.

A comprehensive report on human stool sample handling by Burz et al. demonstrated that for fresh samples, the effect of temperature and atmospheric conditions varied depending on the donor, but storage for >24 h at 37 °C resulted in significant changes; the genera *Lactobacillus, Enterococcus, Ruminococcus 2*, and *Eubacterium* proliferated, whereas many *Ruminococcaceae* family members, such as *Ruminococcus 1* and *Faecalibacterium*, became depleted^109^. Several genera from the *Ruminococcaceae* and *Lachnospiraceae* families had revivification alterations as early as just after 24 h of storage at 37 °C, which is critical considering that these two, along with *Prevotellaceae*, are among the most expanded following FMT in clinical studies^109–112^. Moreover, fresh stool preparations only contain ~75% viable bacteria, with these numbers dropping to only 30% after cryopreservation, irrespective of the duration^109^. Another group indicated that freezing of human feces significantly elevated the Firmicutes to Bacteroidetes ratio when compared with fresh samples, although it is necessary to note that freezing was performed only at −20 °C^113^. In a mouse study, the transplantation of frozen human fecal samples into mice led to an initial low diversity and greater levels of *Erysipelotrichi* compared to colonization with fresh samples, but these levels soon normalized after 7 days to approximate the donor composition^36^. In clinical studies, the efficiency of FMT is often measured by the *C. difficile* clearance rate, as the transplantation of the gut microbiota of a healthy donor leads to pathogen elimination^11,12^. Comparisons on the outcomes of using either fresh or frozen samples have mixed results; some studies have indicated higher pathogen clearance after using fresh samples over frozen samples, whereas others obtained contradicting results (Table 3)^114–117^. However, the contrasting results may be due to many variances between studies, such as the route of transplant delivery, study size, severity of patients’ conditions, and donor pool. Overall, research groups seeking to utilize human fecal samples for FMT in mice should attempt to directly use fresh samples or samples prepared in maltodextrin-trehalose solutions stored at −80 °C before rapid thawing at 37 °C to preserve maximum flora resemblance^109^. This is also in line with the 2017 and 2019 International consensus conferences on approved FMT procedures for clinical application^118,119^.

**Table 3:** Comparison of clinical FMT study methods and outcomes.

Study	Sample state	Sample preparation	Delivery method	Study size	*C. difficile* clearance rate after first treatment
Van Nood et al., 2013^120^	Fresh	Dilution with 0.9% NaCl Supernatant strained Used within 6 h of donation	Nasoduodenal tube	16	81%
Youngster et al., 2014^121^	Frozen at −80 °C	Under aerobic conditions Dilution in NaCl through a blender Sieving and centrifugation before resuspension in NaCl with 10% glycerol 1.6 g aliquoted into size 0 capsules and then into size 00 capsules before freezing at −80 °C Thawed at −20 °C before consumption within 2 h	Capsule consumption	20	70%
Lee et al., 2014^137^	Fresh	Fresh samples homogenized in 300 ml of water with a disposable spatula before 100 ml was transplanted	Retention enema	94	47.9%
Lee et al., 2016^114^	Fresh vs Frozen at −20 °C	Fresh samples diluted in fresh water and emulsified with a wooden spatula Strained through gauze Fresh samples stored at 5 °C before direct usage within 24 h or freezing at −20 °C Frozen samples thawed at 25 °C overnight and used within 24 h	Enema administration	232	52.8% for frozen, 50.5% for fresh
Satokari et al., 2014^115^	Frozen at −80 °C	Fecal suspension in 0.9% NaCl 10% glycerol added 30 g of feces aliquoted and frozen at −80 °C Thawed at room temperature for 4–5 h or at 37 °C	Lower endoscopic delivery into cecum	26 patients for fresh, 23 Patients for frozen	96% for both fresh and frozen
Kassam et al., 2012^122^	Fresh	150 g of fresh sample emulsified in 300 ml of sterile water prior to immediate delivery	Rectal enema	27	81%
Hamilton et al., 2012^116^	Fresh and frozen at −80 °C	50 g of fresh sample diluted in 250 ml of saline water and homogenized in a blender under anaerobic conditions Strained through laboratory sieves Centrifuged and resuspended in saline Used immediately as fresh transplant or frozen at –80 °C with 10% glycerol and thawed for 2–4 h in ice bath	Colonoscopy biopsy channel into terminal ileum and cecum	12 patients for fresh, 21 patients for frozen	92% for fresh, 90% for frozen
Jiang et al., 2017^117^	Fresh, frozen, and lyophilized	50 g of fresh samples processed within 4 h of collection in 0.85% NaCl and mixed in a Stomacher 80 Master (Seward Laboratory System Inc., Davie, FL, USA) before filtering through gauze Fresh samples used within 2 h of preparation Frozen samples stored at −80 °C Lyophilized samples frozen at −80 °C for 6 h before processing in a Freeze Dry System (Labconco, Kansas City, MO, USA) and stored at 4 °C	Colonoscopic delivery into proximal colon	25 fresh sample recipients, 24 frozen sample recipients, 23 lyophilized sample recipients	100% for fresh, 83% for frozen, 78% for lyophilized
Rohlke et al., 2010^138^	Fresh	Fresh sample suspended in saline with manual shaking in a suction canister before filtering and delivery	Colonscopic delivery into the ileum	19	95%
MacConnachie et al., 2009^139^	Fresh	30 g of fresh sample in 0.9% saline Homogenized in a blender and filtered before transplanting 30 ml	Nasogastric delivery	15	73%

Donor health screenings are required in creating a humanized murine gut microbiota model using human transplants to identify any underlying conditions that may affect the gut microbiota composition of the donor. In clinical settings, donors are selected partly through an initial screening using questionnaires and other examinations for the presence of antibiotics, parasites, enteropathogenic bacteria, antibodies for HIV, human T-cell lymphotropic viruses, and hepatitis^115,116,120–122^. However, these questionnaires that are typically utilized often do not address other behavioral patterns of donors that could affect their gut microbial composition, including alcohol consumption, smoking, sleeping patterns, and diet. For research purposes, human fecal samples can come directly from specified individual donors or from a universal source of volunteers; for example, a stool bank. Research groups should plan carefully as to how and where their samples are sourced from and take into account the method of sample preparation utilized, as these may alter the flora colonization in the mouse model.

Routes of delivery for FMT also affect the efficiency of transplant colonization. For human patients, lower endoscopic delivery of FMT is far more effective in pathogen clearance than nasogastric delivery, indicating better microbial uptake (Table 3)^123^. A comparison of FMT procedures between two hospitals in England nicely illustrated these findings, with colonoscopic deliveries and fresh sample usage correlating with higher cure rates as opposed to nasojejunal or frozen stool sample usage^124^. FMT in preterm pigs also supports rectal administration over oral gavage, with the added benefit of reduced sepsis occurrence^125^. However, currently in most research and commercial murine settings, FMT is conducted predominantly through oral gavage, perhaps owing to ease and convenience, as rectal administration further requires colon lavage prior to administration^23,95,126^. Although FMT through oral gavage in mice does successfully confer microbe colonization, it is yet unclear whether rectal administration may be more effective and replicable in mice, invoking the need for comparative studies in GF mice.

### Optimizations for GF mice: husbandry-related factors

**Fig. 1:**
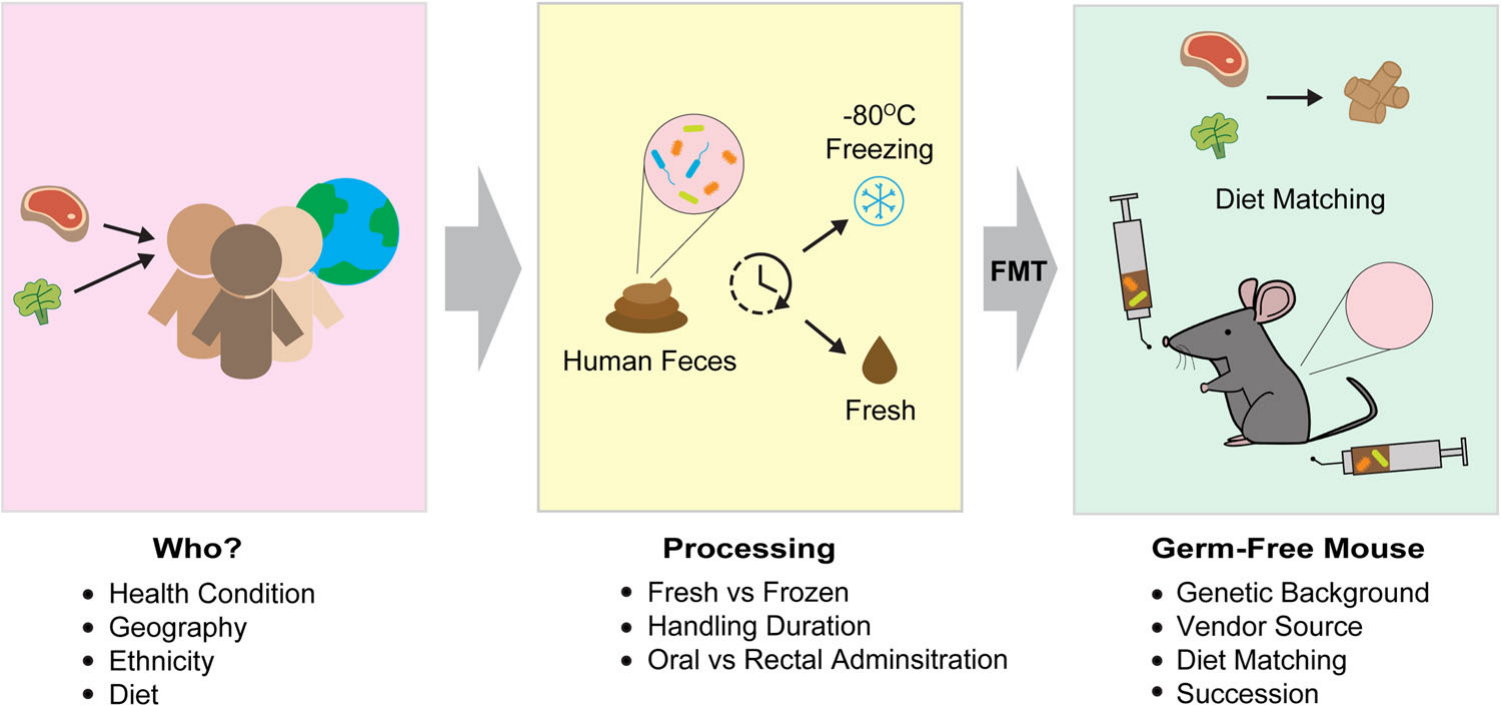
Schematic diagram of creating a humanized gnotobiotic mouse model. In creating a relevant, effective, and reproducible humanized gnotobiotic mouse model, several key factors must be taken into consideration, including form whom the donor fecal samples are collected from, how the samples are processed, and which mouse is selected as the recipient. Optimization for matching the mouse feed to the donor’s diet will also be necessary to ensure efficient continuation of the donor’s microbiota within the mouse setting.

As described above, there are numerous sources for incongruencies in creating a humanized gnotobiotic mouse model, and thus, various points of optimization are necessary for reproducible results (Fig. 1). One such fundamental area is husbandry-related factors that are capable of altering the transplanted human gut microbiota in GF mice. The generation and maintenance of GF mice require significantly more attention than other murine settings, as they mandate specialized facilities, skills, and costs^3^. Vendors of GF mice can help give a small look into the extensiveness of these necessities.

Currently, the two major global vendors for GF mice are Taconic Biosciences and Charles River Laboratories. Taconic Biosciences, which offers GF C57BL/6, BALB/c, Swiss Webster, *IL-10*^*−/−*^ BALB/c and C57BL/6, as well as custom rederivation of donor animal services, defines GF health standards as “no detectable organisms (viruses, parasites, bacteria, fungi)” that are raised in gnotobiotic film isolators^127^. The GF health standard for Taconic utilizes the International Health Monitoring System (IMHS) health testing standard, which includes the “Core Testing Program” that uses sentinels for virus detection, full bacteriology, and parasitology on a quarterly or annual basis, depending on the bacterial or viral agent being tested^128^. In addition, Taconic’s GF mice undergo the Ancillary Microbial Monitoring program, which is made of two parts, the “Isolator Microbial Monitoring” that uses a culture-based screening to check for bacteria and fungi from fecal samples as well as feed, water, and isolator surfaces on a weekly basis from GF isolators, and the “16 S Bacterial RNA Screening”, which uses 16 S PCR of pooled fecal samples in GF isolators on a monthly basis^128^.

At Charles River Laboratories, GF mice are also raised in sterile isolators with autoclaved food and bedding and are screened weekly for bacteria and fungi^129^. Feces and environmental swabs are tested in aerobic and anaerobic cultures as well as phase microscopy for motile organisms, whereas a more comprehensive 16 S PCR bioanalysis is performed quarterly for fecal samples^129^. In addition, necropsy examinations are performed annually to check animal tissues and organs for manifestations of diseases, and blood is screened for pathogen-specific antibodies using multiplexed fluorometric immunoassay and immunofluorescence assays^129^.

Through vigilant screening programs such as those implemented by Taconic Biosciences and Charles River Laboratories, research institutions with in-house GF facilities can achieve normalized microbe-free GF murine models that are latitudinally comparable with other research groups. Therefore, the differences between the gut microbiota of conventional mice purchased from different commercial vendors should not be an issue^130^. However, as seen under non-GF conditions, the composition of the rodent diet, the methods used for sterilizing the diet, the type and frequency of bedding changes, and the stress induced by the exposure of chemical sterilant upon the exchanges of materials inside GF isolators should be taken into consideration in studies of humanized gnotobiotic mice^97^.

### Optimization of humanized gnotobiotic mice: diet matching

As discussed earlier, the gut microbiota compositions of human donors are dependent upon the diet. Upon transplantation into GF mice, microbial species of the human microbiota are exposed to a dissimilar diet, which may cause the gut flora of the murine model to no longer be an exact representative of the human donor. Indeed, Turnbaugh et al.^36^ clearly demonstrated how following FMT of human feces into mice, the feeding of a high-fat, high-sugar “Western Diet” severely disrupts the gut flora, especially when compared with mice on a low-fat, plant-polysaccharide-based diet. A recent study further championed this phenomenon by transplanting obese or lean human fecal samples into mice before feeding a control, 45% high-fat diet-induced obesity, or a “Total Western Diet” that mirrors the American diet^131^. Irrespective of the body type of the human donor, the murine gut microbiota composition was overcome by the diet type, suggesting that the experimental diet has a greater impact than the donor source^131^. The two studies above illustrate two essential ideas. First, for those seeking to evaluate the effects of diet on the microbiome, the data by both Turnbaugh et al. and Rodriguez et al. may indicate that the source of the human fecal sample may not even be relevant. Second, for projects that aim to study the microbiome of one certain population and maintain as much of a similar gut composition within the mouse setting, it will be integral to use a matched humanized mouse diet.

The most commonly used laboratory animal diet is the grain and cereal-based “normal chow” diet, which has been used as the global standard since the 1940s and is composed of unrefined ingredients, such as ground corn, wheat, oats, alfalfa meal, soybean meal, and animal fat, with added micronutrients^132^. One continued concern has been the secretive nature of the exact compositions of chow recipes across manufacturers and the alterations of formulas over time, causing a huge potential for disparity and irreproducibility across studies, especially when considering the sensitivity of the gut flora to diet composition. Furthermore, these diets can often contain pollutants from ingredient sources, including phytochemicals, heavy metals, and lipopolysaccharides endotoxins^132–135^. For this reason, many manufacturers advertise the usage of “purified ingredients” in which each nutrient is derived from highly refined singular sources, allowing purity, vast customizations, and specific source identification for every component of the diet. These products allow modern researchers to create feed recipes that meticulously match the diet of human microbiota donors to attempt to seamlessly maintain the flora across species as much as possible. The diets of humans across regions vary immensely, and the dietary compositions between mice and humans are even greater, giving leeway to tremendous consequences for the development of transplanted microbiota^136^. However, by employing diet questionnaires to fecal donors and customizing corresponding research diets, there is an opportunity to take humanized murine microbiota studies to higher precision and translatability.

## Conclusion

Beyond its relevance for clinical therapies, FMT has become an incredibly central tool in gut microbiota research in the modern era. By taking the human gut microbiota and transplanting it into mice, it is now possible to create high-fidelity replicates of the human gut flora in an experimental system. This powerful mechanism allows precise insight into the inner workings of gut microbes across various diseases and physiological settings, highlighting their importance in human health. However, several factors hinder the seamless transition of transplants between the human and murine guts. In this review, we have outlined the key considerations researchers should take when designing humanized gnotobiotic mouse models.

The basal microbiomes of humans and mice differ, with variations in the abundance of key phyla and the presence of several unique genera. This may be due in part to the anatomical and physiological characteristics, as mice lack appendixes and have a relatively larger colon and cecum than humans, but we believe that the diets and environmental exposures between humans and laboratory mice are even more significant sources of variation. When composing a humanized gnotobiotic animal model, the continuance of the donor microbiota may require the researcher to match the animal diet to the human donor’s diet using purified ingredients. This mode of diet matching will also inevitably raise the question of from whom is the human feces collected. Sufficient evidence points to the compelling distinctions in microbial signatures across dietary modes, geography, age, socioeconomic and health status, and ethnicity, raising a serious question as to whether studies conducted using distinct donor sources are longitudinally comparable. This points to a need for a standardized system for devising humanized gnotobiotic mouse models that are reproducible and translatable among research groups.

We propose the formulation of careful selection criteria for the screening of human fecal donors that allow researchers to, as closely as possible, match the mouse husbandry conditions to those of human donors (Fig. 1). This requires not only the consideration of who the donor is, but also which mouse system will become the recipient, as mouse strains have unique microbial signatures. Progress has already taken place in comparing the effects of some of these variables on GF systems, but for a complete normalization to be possible across institutions, further work is required to analyze the ins and outs of designing humanized models so that a guideline can be formed. With the collaboration of researchers around the world, as well as the help of commercial research industries, it will soon be possible to create fully competent humanized microbiome mouse models that can closely mimic the human system, marking a new era of biomedical progress.
